# Editorial: Medical Application and Radiobiology Research of Particle Radiation

**DOI:** 10.3389/fpubh.2022.955116

**Published:** 2022-07-22

**Authors:** Fei Ye, Chao Sun, Yi Xie, Bing Wang, Lu Cai

**Affiliations:** ^1^Institute of Modern Physics (CAS), Lanzhou, China; ^2^National Institute of Radiological Sciences, National Institutes for Quantum Science and Technology, Chiba, Japan; ^3^Department of Pediatrics, Radiation Oncology, Pharmacology and Toxicology, Pediatric Research Institute, University of Louisville School of Medicine, Louisville, KY, United States

**Keywords:** particle therapy, radiation protection, radiation breeding, personalized medicine, BNCT

Applications of particle radiation technology in the medical and biological fields are focused on disease treatment, radiation protection and radiation biology research related to plants and microorganisms. With the continuous improvement of cancer treatment technologies such as protons, heavy ions and boron neutron capture therapy (BNCT) (1), more and more medical centers have installed and used related equipment in recent years (2). However, due to the different policies of the medical industries, the development speed of particle therapy in various countries varies significantly. Charged ion therapy could offer advantages of biological and physical dose distributions, and higher relative biological effectiveness over conventional photon therapy for treatment of X-ray resistant and hypoxic tumors (3). BNCT, on the other hand, is the hope of radiation therapy for diffuse or irregularly shaped tumors (4). Improving the accuracy and efficiency of treatment remains major challenges for medical physicists (5).

Meanwhile, the worst nightmare for radiation oncologists was the accelerated metastasis and spread of tumors after radiation therapy (6). In addition, although the doctor will make a radiotherapy plan for physical dose delivery for each patient before radiation therapy (7), personalized medicine based on the radiation response of individual patients will be the future trend. The treatment based on individual radiation response now has two promising directions, combination of radiation therapy with immunotherapy and combination of radiation therapy with metabolic therapy.

Radiation protection has been a top priority ever since the recognition of the harmful effects of radiation on the human. For particle radiation protection, the most important research object is the radiation of high-energy charged particles in space.

Although gene editing technology (8) has developed rapidly in the past decade, radiation breeding is still an important way to cultivate crop seed lines, especially the development of particle accelerator technology and the continuous deepening of aerospace research, which has made the research of particle radiation breeding more in-depth.

In this Research Topic, we invited scientists studying high-energy charged particles either for cancer treatment or for radiobiology research. The Research Topics accepted 15 articles including a total of 92 authors, demonstrating the high interest in several of the topics described above. As of this writing, the Research Topics have been viewed over 25000 times. The articles can be categorized into the following topics.

## Medical physics technology

Four articles are dedicated to the improvement of dose delivery accuracy and treatment efficiency in particle therapy. In a highly viewed article, Li et al. reported a beam shaping assemblies (BSAs) design for accelerator based BNCT with multi terminals. The design of this beam shaping assembly allows one proton accelerator to be used to produce both epithermal and thermal neutrons. The latter is suitable for the treatment of superficial tumors, such as melanoma, and for preclinical experiments in small animals. The combination of MRI and proton therapy system transforms the traditional radiotherapy process (localization-treatment) into an adaptive workflow (MRI scan-plan optimization-treatment implementation), to allow timely adjustment of the treatment plan according to tumor volume changes and realizes real-time individualization of each fractionation for each patient, which is important for improving tumor control rate and reducing toxic side effects. Proton trajectories have deflection in the magnetic field, and the paper by Wang et al. calculates the dose deviation in the dose calculation of the proton therapy system when combined with MRI. PET can detect the induced radioactivity of charged particle streams *in vivo* to monitor information such as Bragg peak location and dose distribution. Zhang et al. found that off-line PET can detect ultra-low radioactivity down to 30 Bq/ml, this property allows off-line PET to be used to evaluate the completion of particle treatment plans. In the traditional radiotherapy workflow, organ segmentation depends heavily on physician expertise (9). Shen et al. automatically segmented 17 human organs using a neural network algorithm, which improved the accuracy and speed of organ segmentation.

## Basic research related to cancer treatment

Whether radiotherapy promotes tumor metastasis is a controversial topic. In this issue, two studies on carbon ion irradiation of gastrointestinal tract cancer cell lines reveal the metastasis inhibitory effect of carbon ions. Si et al. found that carbon ion irradiation not only increased the growth inhibition of CAL27 cell line (tongue squamous cell carcinoma) through FAK signaling pathway, but also decreased the metastatic ability of cancer cells. Luo et al. found that carbon radiation inhibited the metastatic ability of ECA109 and KYSE150 cell lines (esophageal squamous cell carcinoma) by modulating the JAK2/STAT3 signaling pathway. In addition, Ma reviewed the research progress on the anti-metastatic effects and mechanisms of photon or particle beam radiotherapy combined with immunotherapy, followed by the prediction of the future of this research area. Regarding individualized medicine in radiotherapy, currently it seemed only applied in the customization of dose delivery schedules. Apparently, it is a limitation because differences in metabolic capacity and levels of immune activation among different individuals may make the same treatment plan with different prognoses. In fact, this notion is supported by the following studies under different conditions. Ning et al. investigated metabolic end products in the urine of prostate cancer patients treated with carbon ion radiation, and they found that metabolic reprogramming and inhibition are involved in response to carbon ion radiotherapy in patients with prostate cancers. Therefore, they suggested the potential application of examining urine metabolites timely to assess individual response to carbon ion radiotherapy. Using single cell transcriptome technology to compare immunogenic molecular markers produced after X-ray and carbon ion irradiation of lung cancer cell lines, Ran et al. revealed a stronger immune response of these cancer cells in response to carbon ion irradiation than X-ray.

It is well-known that the COVID-19 pandemic has significantly disrupted the normal treatment of patients with cancer. Li et al. have re-evaluated the safety and efficacy of carbon ion radiation therapy for unresectable liver cancer and also compared with transarterial chemoembolization under such unexpected and undesirable condition. Consequently, carbon ion radiation therapy was able to achieve superior overall survival, local control, and relative hepatic protection since it can maximize the utilization of inpatient and outpatient treatment for patients with unresectable liver cancer due to shorter hospital stays (due to a shorter treatment course) and reduced care needs (due to low normal tissue toxicity). In addition, carbon ion radiation therapy allows in-hospital telemedicine to maintain sufficient person-to-person physical distance throughout the treatment of unresectable liver cancer, which is significant for cutting off the transmission route of the virus.

## Particle radiation protection

In addition to artificial manufacturing, particle radiation is generally found in radon gas and in space. By comparing data from space missions and ground-based experiments, Bartoloni et al. compiled dose-effect relationships for several particle radiation health risk events. In a ground-based experiment simulating a space environment, Ma et al. evaluated the effects of proton radiation combined with microgravity effects on mouse embryonic osteoblast precursor cells (MC3T3-E1) and showed that cell proliferation and differentiation capacity were reduced in a dose-dependent manner. After demonstrating the protective effect of transcription factor P53 modulators against low-energy X-ray or gamma radiation-induced damage, Morita et al. further demonstrated that P53 modulators were also effective in protecting the hematopoietic system and mitigating intestinal damage during high-energy carbon or iron ion beam irradiation.

## Others

Food supply is of common international concern. With increasing populations and rapid climate change, increasing food production has become an imperative. Although advanced technologies such as gene editing provide an effective way to breed varieties, radiation is able to mutate to improve germplasm diversity because it can bring about more random mutations throughout the genome. Ma et al. reviewed the achievements and progress of traditional photon radiation breeding and described the developments in seed production research brought by particle radiation based on particle accelerator and cosmic radiation received on space stations that have emerged in recent years.

Particle accelerator is the largest medical device, which is expensive and occupies a large area, and the industrialization process of particle therapy is greatly influenced by the industrial policy of each country. Dai et al. shared the industrial policy of particle therapy in China and the difficulties encountered in the industrialization process. This article has the highest number of views, which indicates that readers have a strong interest in this topic.

In summary, this special issue reviews past and current works, and provides new findings in researches and applications related to particle radiation, with an emphasis on the particle therapy in cancer treatment. We also collected articles on topics related to particle radiation protection, radiation seeding and the industrialization of particle therapy devices. We hope that this special issue could present the readers with a big picture of particle radiation applications and bring new ideas to researchers.

## Author contributions

FY drafted the manuscript. BW, CS, and YX revised the manuscript. LC finalized the manuscript. All authors contributed to the article and approved the submitted version.

## Conflict of interest

The authors declare that the research was conducted in the absence of any commercial or financial relationships that could be construed as a potential conflict of interest.

## Publisher's note

All claims expressed in this article are solely those of the authors and do not necessarily represent those of their affiliated organizations, or those of the publisher, the editors and the reviewers. Any product that may be evaluated in this article, or claim that may be made by its manufacturer, is not guaranteed or endorsed by the publisher.
